# Disseminated Histoplasmosis as a Presentation of Advanced HIV-1 Infection in a Non-endemic Country

**DOI:** 10.7759/cureus.78581

**Published:** 2025-02-05

**Authors:** Débora A Alves, João Trêpa, José Sousa-Baptista, João Pereira-Vaz, Rui Tomé

**Affiliations:** 1 Infectious Diseases Department, Centro Hospitalar e Universitário de Coimbra, Unidade Local de Saúde de Coimbra, Coimbra, PRT; 2 Intensive Care Unit, Unidade Local de Saúde de Viseu Dão-Lafões, Viseu, PRT; 3 Clinical Pathology Department, Coimbra Local Health Unit, Coimbra, PRT

**Keywords:** histoplasma capsulatum, histoplasmosis, hiv/aids, infections in immunocompromised patients, non-endemic country

## Abstract

Disseminated histoplasmosis is an opportunistic fungal infection considered an AIDS-defining illness, sometimes with a fatal outcome. We report a case of disseminated histoplasmosis as the initial presentation of advanced HIV-1 infection in a migrant living in a non-endemic area. Histoplasmosis was suspected based on the clinical presentation, epidemiological factors, and observation in the peripheral blood smear of neutrophils and monocytes with yeast-like forms. The diagnosis was confirmed through a positive peripheral blood smear and subsequent culture of *Histoplasma capsulatum* from a bronchoalveolar lavage sample. The patient was treated with 14 days of liposomal amphotericin B plus oral itraconazole for two years, with a good response. Direct examination of peripheral blood has been considered a valuable diagnostic method, specifically in severely immunocompromised patients, as our case report. The presented case highlights the importance of considering disseminated histoplasmosis in the differential diagnosis of HIV-infected patients, even in non-endemic regions. Hence, the need for a high index of suspicion to ensure early diagnosis and treatment is crucial as the influx of migrants increases globally.

## Introduction

*Histoplasma capsulatum*, which comprises two varieties, var.* capsulatum* and var.* duboisii*, is the etiological agent of histoplasmosis. The *H. capsulatum* has soil, bat, and bird waste as its natural reservoir, where it adopts a sporulating mould made up of a network of hyphae [1,2]. The infection occurs through inhalation of spores in contaminated fomites. Inside the host (at 37ºC), the sporulating forms give rise to yeast cells that invade cells [1].

Although found worldwide, var.* capsulatum* is considered endemic to the American continent and var. *duboisii *to the African continent. Nevertheless, in recent decades, cases of histoplasmosis have been described in Europe and Asian countries such as China, where this infection is not considered endemic [1]. The immigration of people from endemic countries such as South America, particularly Brazil, and the African continent has also contributed to the upsurge in cases in Portugal [3,4].

Histoplasmosis can present in a broad spectrum of clinical forms, ranging from asymptomatic, self-limiting or localised infection to severe disseminated disease. The manifestations largely depend on the individual's immune status and the magnitude of the inhaled inoculum [5]. In immunocompetent individuals, histoplasmosis often causes no symptoms or mild, flu-like illnesses. Some may develop more severe respiratory problems, like pneumonia or chronic lung disease, especially those with existing lung conditions. In immunocompromised individuals, such as those with AIDS, histoplasmosis is more prone to disseminate throughout the body [6]. The infection can spread from the lungs to other organs, including the liver, spleen, central nervous system, bone marrow, and adrenal glands [5]. Disseminated histoplasmosis can be life-threatening if left untreated. Symptoms may vary depending on the organs affected but can include fever, weight loss, fatigue, hepatosplenomegaly, lymphadenopathy, and skin rash. Central nervous system involvement may lead to meningitis or encephalitis. Histoplasmosis can be easily mistaken for other conditions, such as tuberculosis or lymphoma, particularly in regions where these diseases are more prevalent [7]. Therefore, maintaining a high index of suspicion is crucial for prompt diagnosis and appropriate management.

The gold standard for diagnosis is the isolation of* H. capsulatum* through culture from clinical samples, like blood, bone marrow, sputum, or tissue biopsies [8]. However, this can be time-consuming, often taking weeks to get results. The microscopic examination in a peripheral blood smear sometimes allows the detection of *H. capsulatum* yeasts in clinical samples, particularly in disseminated disease [9]. Serologic tests, such as immunodiffusion or complement fixation, can detect antibodies against *H. capsulatum,* depending on the clinical form, with a reported sensitivity between 70% and 100% and a specificity close to 100% [4]. Antigen detection is especially valuable in acute diseases, particularly in people co-infected with HIV, as they often exhibit disseminated histoplasmosis without detectable antibodies to the fungus [4]. While these tests can be helpful, they may not be positive in the early stages of infection or immunocompromised individuals. Furthermore, cross-reactivity with other fungal infections can occur (paracoccidioidomycosis, blastomycosis) [4]. Molecular methods, such as real-time quantitative polymerase chain reaction (PCR), have also become promising diagnostic tools, being faster and more sensitive (100% sensitivity). Chest radiography and computed tomography scans can reveal characteristic findings suggestive of histoplasmosis, such as pulmonary nodules, infiltrates, or mediastinal lymphadenopathy. However, these findings are not specific to histoplasmosis and can mimic other respiratory infections (e.g., the miliary pattern may be seen in miliary tuberculosis, histoplasmosis, blastomycosis, silicosis, berylliosis, miliary sarcoidosis, and metastatic malignancy) [10]. 

The diagnostic approach should be tailored to the patient's clinical presentation, immune status, and the available resources in the healthcare setting.

## Case presentation

**Figure 1:**
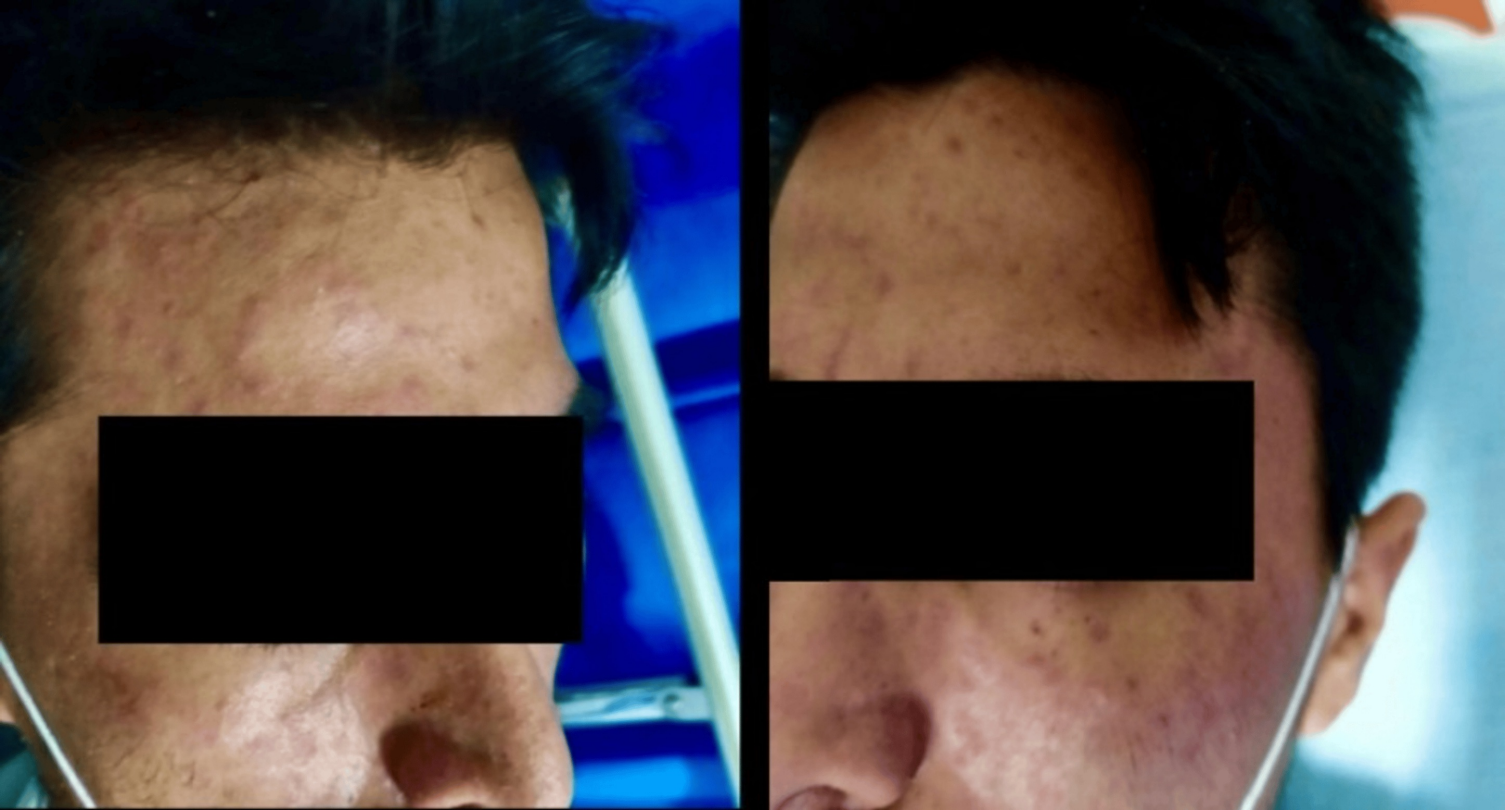
Mild maculopapular rash on the face.

**Figure 2:**
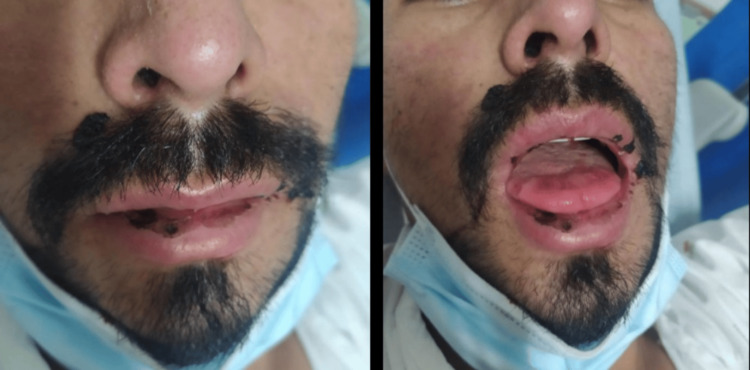
Oropharyngeal candidiasis and oral mucosal ulcerative lesions.

A 30-year-old Colombian man, living in Portugal for the last three years, was admitted to our emergency department (ED) in February 2022 with a one-month history of persistent fever (maximum 40ºC) at the end of the day, weight loss (about 10 kg), sweating, and watery diarrhoea. He also reported experiencing a dry cough, easy fatigue, shortness of breath, and painful swallowing, with no history of nausea or vomiting. The patient had been evaluated two weeks earlier in the ED due to the same symptoms; at the time, he was diagnosed with salmonellosis and treated with ciprofloxacin 500 mg bid for eight days, with temporary improvement of his symptoms. However, his condition worsened following the completion of antibiotics, and he needed to be readmitted to the hospital. He had returned from Colombia three weeks prior to the episode, and his medical history was remarkable for treated pulmonary tuberculosis (in 2019) and non-treated epilepsy. At the ED, he was febrile (tympanic temperature of 40.7ºC) and hypotensive (blood pressure 90/56 mmHg), with a Glasgow Coma Scale (GCS) of 15 and oxygen saturation of 97%. He presented painless lymphadenopathy in the right posterior cervical area and a mild maculopapular rash on his face without pruritus (Figure 1). He also presented oropharyngeal candidiasis and oral mucosal ulcerative lesions (Figure 2).

**Figure 3:**
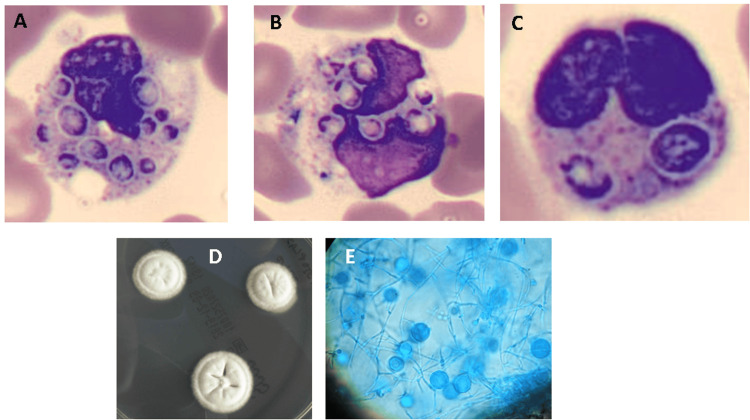
A-C: Peripheral blood smear (May-Grunwald-Giemsa, × 1000) within 7-8% of neutrophils and monocytes yeast-like forms with an eccentric nucleus and an apparent pseudo capsule. Rare similar forms have also been observed extracellular. These visualised forms are suggestive of disseminated Histoplasmosis. The intracellular load was 1 to 10 organisms. D: White colonies with a cottony and sometimes warty appearance observed on the 18th day of culture. E: (Blue lactophenol, x1000) Microscopic exam of the colonies showing thick-walled single-celled macroconidia.

Laboratory results revealed pancytopenia, acute kidney failure (AKIN 3) with compensated metabolic acidosis without elevated lactate (blood gas analysis: pH 7.36, pO_2_ 81 mmHg, pCO_2_ 26mmHg, lactates 1.5 mmol/L, HCO_3-_ 14.7mEq/L), severe cytocholestasis, and elevated inflammatory markers (Table 1). A chest radiograph showed bilateral basal infiltrates, and an abdominal ultrasound noted a hepatomegaly of 17 cm with no other abnormalities. Blood cultures and urine antigen tests (for *Legionella pneumophila, Streptococcus pneumoniae,* and *Histoplasma*) were collected, and the patient was started on empiric ceftriaxone and azithromycin, as well as fluids. An Infectious Diseases (ID) consultation was requested in the ED, and additional tests were performed. HIV serology was reactive for HIV-1, with an HIV RNA viral load >10,000,000 copies/mL and a low CD4+ T lymphocyte count of 32 cells/mm^3^ (10.4%). Acid-fast bacilli smears were negative. Considering the patient's epidemiology, histoplasmosis was suspected. Seeing his multi-organ dysfunction and despite responding to fluids, he was admitted to the intensive care unit (ICU) for the management of sepsis. A peripheral blood smear revealed intracytoplasmic yeast cells consistent with *H. capsulatum* (Figure 3). The patient was then started on targeted antifungal therapy with liposomal amphotericin B (5 mg/kg/day). At the laboratory, blood was inoculated in Sabouraud/chloramphenicol-agar plates and incubated at 25ºC and 37ºC. Characteristic colonies and macroconidia of *Histoplasma* were observed, as described in Figure 3. DNA Sanger sequencing was performed to achieve a more accurate identification. The combination of the highest identity, total score and query values identifies the species as a *H. capsulatum.* The definitive diagnosis was subsequently confirmed by the cultural isolation of *H. capsulatum* from a bronchoalveolar lavage (BAL) sample and a positive *Histoplasma* urinary antigen (2022).

During the patient's stay in the ICU, he was also diagnosed with *Pneumocystis jirovecii* pneumonia and, therefore, started on trimethoprim/sulfamethoxazole (adjusted to renal function) and corticosteroid therapy. Lip biopsy was positive for type 1 herpes simplex infection. In addition, oral nystatin, acyclovir and antiretrovirals (emtricitabine/tenofovir + dolutegravir) were also prescribed. The patient evolved favourably, gradually improving his organ dysfunction. The table shows the evolution during hospitalisation (Table 1).

**Table 1:** Patient's analytical evolution during hospitalisation MCV: mean corpuscular volume; INR: international normalized ratio; PT: prothrombin time; APTT: partial thromboplastin time; LDH: lactate dehydrogenase; AST: aspartate transferase; ALT: alanine transaminase; GGT: gamma-glutamyl transferase; CK: creatine kinase; PCT: procalcitonin

Blood tests/day of hospitalisation	Reference values	Day 1 hospital admission	Day 3	Day 5	Day 8	Day 30 hospital discharge
Haemoglobin (g/dL)	13.5-17.5	11.1	9.3	7.2	8.3	9.6
MCV (fL)	80-100	-	78.5	79.8	82.3	84.0
Leukocytes (x10^9/L)	3.90-10.2	3.3	4.6	9.5	4.3	5.2
Lymphocytes (x10^9/L)	1.10-4.50	0.29	-	-	-	0.66
Platelets (x10^9/L)	150-450	133	127	81	53	456
INR	0.8 - 1.2	1.13	0.99	-	1.16	-
PT (sec)	9.4 - 12.5	13.1	11.5	-	13.5	-
APTT (sec)	23.4 - 35.4	38	30.7	-	24.1	-
Creatinine (mg/dL)	0.72 - 1.18	4.18	4.13	1.93	1.12	0.92
LDH (U/L)	< 248	7094	5382	2826	628	132
AST (U/L)	< 35	1046	861	265	54	19
ALT (U/L)	<45	197	191	115	82	54
Alkaline phosphatase (U/L)	30 - 120	476	797	746	475	282
GGT (U/L)	< 55	330	715	644	353	137
Total bilirubin (mg/dL)	0.2 - 1.2	0.7	1.2	0.5	0.6	0.4
CK (U/L)	< 171	82	65	42	11	16
C-reactive protein (mg/dL)	< 0.50	31.05	17.52	10.7	1.16	0.06
PCT (ng/mL)	0 - 0,5	96.3	-	-	1.85	-

The patient completed 14 days of intravenous liposomal amphotericin B treatment, followed by a transition to oral itraconazole 200 mg twice daily on the 15th day of hospitalisation. This oral itraconazole regimen was continued until the *Histoplasma* urinary antigen test became negative (2024). He also completed 21 days of therapy for pneumocystosis with trimethoprim/sulfamethoxazole, maintaining a secondary prophylaxis regimen (double-strength tablet dose) for another 16 months, until CD4+ T lymphocytes count >= 200 cells/mm^3^ for >3 consecutive months. Newly onset fever with no infection foci developed after suspending corticosteroid therapy and with a notable recovery in CD4+ T lymphocytes count from 32 to 161 cells/mm^3^ (10.4-25.3%), and an undetectable HIV viral load was interpreted as an Immune reconstitution inflammatory syndrome (IRIS), so methylprednisolone was restarted on a slow weaning schedule. At that time, the patient had been using antiretrovirals (emtricitabine/tenofovir + dolutegravir) for 20 days. Upon discharge, after a 31-day hospitalisation, the patient was hemodynamically stable, without recurrence of fever, respiratory failure, or other persistent symptoms and normal kidney and liver functions. Outpatient follow-up revealed sustained virological response to antiretroviral therapy (high adherence to the regimen), with undetectable HIV viral load, increasing CD4+ T lymphocyte count and complete resolution of histoplasmosis and other opportunistic infections.

## Discussion

In Portugal, a study performed between 2009 and 2015 reported 10 cases of histoplasmosis in immunocompromised patients, primarily diagnosed in the Lisbon and Tagus Valley region [1]. The study reported five cases caused by *H. capsulatum* var. *capsulatum*, four of which involved disseminated disease. Two cases occurred in HIV-infected patients, and three were in kidney transplant recipients. Most patients were from Portugal, one from Brazil, and one from Congo. However, since histoplasmosis is not a notifiable disease in Portugal, these documented cases were likely diagnosed during hospital admissions [1,4]. As such, the true burden of histoplasmosis in Portugal remains unknown.

A five-year European study reported 118 cases of histoplasmosis between 1995 and 1999, with locally acquired cases reported in Germany, Italy, and Türkiye [11]. A comprehensive review of histoplasmosis cases reported in Europe and Israel from 2005 to 2020 identified a total of 728 cases across 17 European countries [12]. The majority of cases were imported, primarily from Central and South America. However, there were also seven locally acquired cases, six of which were in Europe and one in Israel. In Europe; cases of the disease are typically imported by migrants and travellers, although there have been some instances of locally acquired infection, notably in Italy. However, a significant proportion of European physicians lack familiarity with the clinical and pathological manifestations of the disease, which may indicate underreporting or underdiagnosis of cases. A further systematic review, undertaken with the objective of informing the World Health Organization's Fungal Priority Pathogens List, comprehensively examined literature from 2011 to 2021 and found a high prevalence (22-44%) of histoplasmosis in people living with HIV, with mortality rates ranging from 21% to 53% [13]. Despite the paucity of data available, it is reasonable to infer that the prevalence of histoplasmosis remains stable [13]. 

Disseminated histoplasmosis in HIV-infected individuals has been classified as an AIDS-defining illness since 1987. However, such cases are often underestimated, particularly in tropical regions. Fever, a common symptom of disseminated histoplasmosis, may be mistaken for other endemic tropical infections, such as malaria, tuberculosis, or HIV infection itself [14]. The clinical manifestations of disseminated histoplasmosis in immunocompromised patients with high persistent fever, fatigue, and weight loss, accompanied by hepatosplenomegaly and lymphadenopathy, resemble closely disseminated tuberculosis [14]. Immunocompetent individuals, particularly those engaged in professional activities involving or in close proximity to contaminated material with this dimorphic fungus, are susceptible to developing histoplasmosis. However, it is particularly pertinent to note that those with compromised immune systems are at increased risk of acquiring the disease and experiencing severe forms [1,3]. Among these, the groups at higher risk are individuals with advanced HIV infection or those receiving suboptimal antiretroviral therapy.

Diagnosis often requires the use of multiple methods, such as fungal culture, microscopic examination, detection of *Histoplasma* proteins (histoplasmin, made up mainly of antigens C, H, and M), peripheral blood antibodies, molecular testing, and imaging studies [1,8]. The ability to detect *H. capsulatum* depends on the disease burden and the host's immune status, with a positive diagnosis being more likely in cases of disseminated disease in immunocompromised individuals. Blood cultures, which typically have a low diagnostic yield, exhibit a sensitivity of approximately 50% in patients with advanced HIV, while techniques such as lysis centrifugation can further improve detection rates. In addition, respiratory sample analysis has demonstrated a positivity rate of up to 90%, with higher detection observed in patients with disseminated histoplasmosis (74%) compared to those with acute pulmonary histoplasmosis (42%) [8]. Visualizing yeast forms in peripheral blood is uncommon, and a few case reports have been published [9,13,15-17]. For HIV-infected patients suspected of having histoplasmosis, examining a peripheral blood smear is a vital diagnostic step, although it requires a trained and experienced microbiologist to distinguish it from other infectious agents. The primary applications of serological tests are in the context of chronic diseases and epidemiological studies, and their efficacy in immunocompromised patients is limited. Conversely, the detection of urinary or serum antigens for *Histoplasma* demonstrates a sensitivity of approximately 90% [13], thereby facilitating early diagnosis and serving as a marker of treatment response [3,8].

*Histoplasma* antigen testing is not widely available, and culturing the fungus can take several days (within two to three weeks but may take up to eight weeks) [8]. Even if antigen or antibody tests are not available, the blood smear remains a feasible and rapid diagnostic approach worldwide. Both antigen testing and culture are important to establish the diagnosis, as a negative antigen test does not exclude histoplasmosis [18]. Negative cultures, lack of symptoms, and antigenemia can help distinguish between resolved disease, old disease, and active infection. The most robust evidence for the use of urinary or serum antigens as a treatment response marker is documented in the context of HIV patients. A cut-off value of <2 ng/ml for antigen levels in both urine and serum has been proposed as a prerequisite for discontinuing antifungal treatment [8]. We applied this cut-off value for the discontinuation of antifungal in our patient.

Despite the World Health Organization's inclusion of *Histoplasma* antigen tests on the List of Essential In Vitro Diagnostics, these tests are not routinely available in non-endemic countries [19]. Consequently, diagnosing mycoses can present a significant challenge for clinicians in these regions. For AIDS patients diagnosed with disseminated histoplasmosis through a peripheral blood smear, the outlook is grim without treatment. However, with prompt and effective antifungal therapy along with adherence to antiretroviral medication, the prognosis significantly improves, as we can see in the clinical case presented. The treatment regimen and duration for disseminated histoplasmosis in HIV-1 patients depends on the severity of the infection and the patient's immune status. Severe or life-threatening cases typically start with intravenous amphotericin B, followed by prolonged oral itraconazole for consolidation and maintenance. Close monitoring is crucial to ensure successful treatment and manage any potential side effects from antifungal medications (e.g. nephrotoxicity, electrolyte disorders) [15].

Overall, the key aspects in the management of disseminated histoplasmosis in HIV-1 patients are 1) a high index of suspicion, 2) use of appropriate diagnostic modalities, 3) prompt initiation of antifungal therapy, and 4) long-term maintenance therapy to prevent relapse in immunocompromised patients [18,20].

## Conclusions

Histoplasmosis, an endemic fungal infection, is seldom diagnosed in non-endemic countries like Portugal. Although it is a rare disease in Europe, researchers warn of the need to better understand the epidemiology of this infection at a national level. This case report aims to increase awareness of the diverse clinical presentation of disseminated histoplasmosis, the significant importance of blood smear as an early diagnostic tool, and to highlight the evolving epidemiological pattern of this infection in our country. To this end, it is crucial to enhance knowledge about the pathogenicity, differential diagnostic methods, and early treatment of histoplasmosis and to prevent the progression of systemic infections and the emergence of future cases.
